# Proton-driven sodium secretion in a saline water animal

**DOI:** 10.1038/s41598-024-62974-4

**Published:** 2024-06-03

**Authors:** Marjorie L. Patrick, Andrew Donini, Andrew Zogby, Christopher Morales, Michael J. O’Donnell, Sarjeet S. Gill

**Affiliations:** 1 Department of Biology, University of San Diego, 5998 Alcalá Park, San Diego, CA 92111 USA; 2 Department of Biology, York University, 4700 Keele St, Toronto, ON M3J 1P3 Canada; 3 Department of Biology, McMaster University, 1280 Main St. West, Hamilton, ON L8S 4K1 Canada; 4 Department of Molecular, Cell and Systems Biology, University of California, Riverside, 900 University Ave., Riverside, CA 92521 USA

**Keywords:** Physiology, Animal physiology

## Abstract

Aquatic animals residing in saline habitats either allow extracellular sodium concentration to conform to environmental values or regulate sodium to lower levels. The latter strategy requires an energy-driven process to move sodium against a large concentration gradient to eliminate excess sodium that diffuses into the animal. Previous studies of invertebrate and vertebrate species indicate a sodium pump, Na^+^/K^+^ ATPase, powers sodium secretion. We provide the first functional evidence of a saline-water animal, *Aedes taeniorhynchus* mosquito larva, utilizing a proton pump to power this process. Vacuolar-type H^+^ ATPase (VHA) protein is highly expressed on the apical membrane of the posterior rectal cells, and in situ sodium flux across this epithelium increases significantly in larvae held in higher salinity and is sensitive to Bafilomycin A_1_, an inhibitor of VHA. We also report the first evidence of splice variants of the sodium/proton exchanger, NHE3, with both high and low molecular weight variants highly expressed on the apical membrane of the posterior rectal cells. Evidence of NHE3 function was indicated with in situ sodium transport significantly inhibited by a NHE3 antagonist, S3226. We propose that the outward proton pumping by VHA establishes a favourable electromotive gradient to drive sodium secretion via NHE3 thus producing a hyperosmotic, sodium-rich urine. This H^+^- driven Na^+^ secretion process is the primary mechanism of ion regulation in salt-tolerant culicine mosquito species and was first investigated over 80 years ago.

## Introduction

Insects are the only other major group of animals, aside from mammals and birds, that can concentrate their urine to an osmotic value exceeding their blood or hemolymph value as a strategy to conserve water^1^. Terrestrial environments served as a desiccation selection force on mammals and birds to evolve the renal countercurrent mechanism of concentrating urine to effectively retain water. In contrast, terrestrial insects evolved two different urine-concentrating mechanisms involving hindgut organs. Rectal papillae or pads, as found in desert locusts (*Schistocerca*), cockroach (*Periplanenta*) and many dipterans, including adult mosquitoes, establish local osmotic gradients to enable water reabsorption from urine and back into the hemolymph. Cryptonephridial complexes found in *Coleoptera*, *Lepidoptera* and *Hymenoptera* consist of the distal ends of the Malpighian tubules closely associated with the rectum and enclosed within a perinephric membrane. This system can establish an even greater osmotic gradient to pull most of the water from the rectal lumen thus forming a dry pellet or even absorb water vapour from the ambient air. These powerful urine-concentrating mechanisms in terrestrial insects rely upon active ion secretion (KCl, NaCl) and differential water permeability of hindgut epithelia so as to establish localized osmotic gradients that will draw water back into the hemolymph and minimize excretory water loss via the urine^2^.

When saline-water insects belonging to *Diptera*, including larval mosquitoes, were first noted to be hypo-osmoregulators when residing in saline habitats^3^, it was assumed that these insects would possess a hindgut with similar water absorption properties as described above for terrestrial insects^4^. The first studies of saline-water mosquito larvae *Aedes detritus* employed alimentary canal ligation to identify the hindgut as the site of hyperosmotic fluid production^5^. Ramsay^6^, also studying *A*.* detritus*, reported that the preliminary urine generated by the Malpighian tubules prior to entering the rectum was isoosmotic to the hemolymph whereas the rectal fluid excreted via the anus was found to be strongly hyperosmotic and equivalent to or slightly hypertonic to ambient salt-water values. These trends were echoed in several saline-water mosquito species belonging to *Aedes*, *Anopheles*, and *Opifex* genera^7–12^. However, these aquatic insects were not selectively reabsorbing water from the rectal fluid like their terrestrial cousins but rather employing a third mechanism of generating hyperosmotic urine—actively secreting ions into the urine.

Salt-tolerant, osmoregulating insects are similar to terrestrial animals in experiencing a continuous water-loss stress due to their hypoosmotic hemolymph when residing in saline media (> 300 mOsm). To attenuate this osmotic stress, cuticular permeability is reduced in larvae held in saline water compared to larvae residing in more dilute media^13^. Despite this integumental adjustment, larvae must still imbibe the salty water to achieve water balance. *Aedes* mosquito larvae drink the external saline medium at surprisingly high rates (130–240% body volume/day) whether they are feeding or not^14^. The amount of water imbibed can greatly exceed the amount excreted from the rectum demonstrating an effective means to achieve water balance. Salts, however, are also absorbed across the midgut with the ingested water^15^ and represents a substantial salt load in the hemolymph. Since water balance is achieved by drinking, is it the salt stress that served as a selection force for the evolution of a novel rectal segment capable of generating salty urine?

Ramsay^6^ not only was the first to document that salinity tolerance was conferred by the function of the rectum in larval *Aedes detritus* but also that it was morphologically distinct from freshwater species in being a segmented, two-part rectum. Further studies confirmed similar two-part recta consisting of an anterior and posterior segment in *Aedes campestris, detritus, dorsalis*, and *taeniorhynchus* all belonging to the subgenus *Ochlerotatus*^8,16,17^. *Aedes togoi*, a member of the subgenus *Finlaya*, has a one-part rectum but instead evolved an elaborated anal canal^18^. The derived posterior recta and anal canal in these salt-tolerant species all share common morphological features: large cells with well-developed, deep apical membrane folding, studded with particles, and associated with a large number of mitochondria. This pattern departs from that described for the rectal pads of terrestrial insects where the lateral membrane is extensively folded with a large portion of the mitochondrial population in close proximity^16^. Additionally, the posterior rectal features depart from anterior recta of salt-water species and one-part rectum of freshwater obligate species that have smaller cells, less elaborate apical membrane but a more developed basal labyrinth^19^. These disparities in the location of enhanced membrane system implies different functions. Lateral membrane systems are associated with water reabsorption capability as seen in terrestrial rectal systems whereas elaborate apical membranes as seen in larval posterior rectum suggests ion transport enhancement.

Building on the findings described above, the comprehensive studies of Bradley and Phillips^7–10^ brought more clarity to the hypo-osmoregulatory strategy of *Aedes* larval mosquitoes by confirming that active ion secretion into the lumen of the posterior rectum of *A*.* taeniorhynchus* is the means of hyperosmotic urine production. The authors employed in vivo and in vitro preparations of the recta from larvae held at different salinities and measured the electropotential difference and ionic ratios across the rectal epithelium to determine that Na^+^, Cl^−^, K^+^ and Mg^2+^ are actively secreted into the posterior rectal lumen. Additionally, they reported that both rates of Na^+^ secretion from hemolymph to urine and the trans posterior rectal electrical potential quickly increase in response to increased hemolymph (or bathing medium) Na^+^ levels. In the final paper of the series, Bradley and Phillips^10^ proposed that this trend “is consistent with the stimulation of the electrogenic Na^+^ pump” and that “the apical border seems the more likely site of osmotic work on ultrastructural grounds”. In a later review, Bradley^20^ reaffirmed this idea by stating, “powerful ion pumps located in a unique cell type have evolved in the posterior rectal region”.

The goal of this present study was to employ more recent technology to investigate further the above ideas proposed by Bradley regarding how hyperosmotic urine is produced by active ion secretion. Using a combination of protein expression assays and in situ ion flux measurements, we localized two ion transporter proteins and directly measured their function in the active secretion of Na^+^ across the posterior rectum of larval *A*.* taeniorhynchus* in high salinity water. This proton-driven mechanism of sodium secretion that we have characterized is in contrast to the mechanisms evident in all other saline-water invertebrate or vertebrate species^21–23^ and confirms the novelty of this ionoregulatory organ.

## Materials and methods

### Mosquitoes

**Figure 1:**
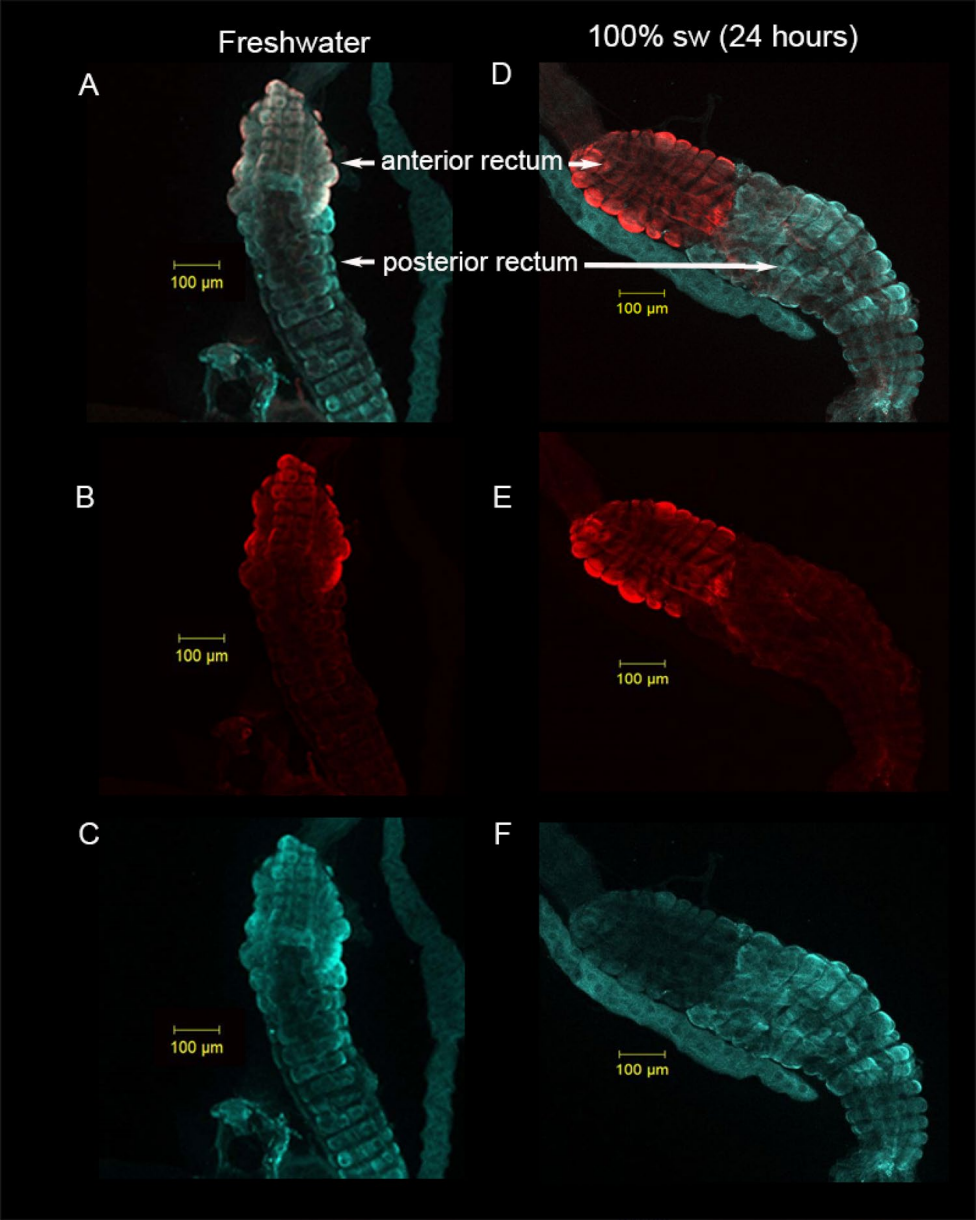
Expression of V-type H^+^ ATPase and P-type Na^+^/K^+^ ATPase protein in rectum of *A*.* taeniorhynchus* larvae. Whole mount of the two-part rectum from larval *A*.* taeniorhynchus* acclimated to 1% (**A**–**C**) and 24 h post-transfer from 1 to 100% seawater (**D**–**F**) showing immunolocalization of V-type H^+^ ATPase protein (Cy5 blue **A**, **C**, **D**, **F**) to both the anterior and posterior rectum. P-type Na^+^/K^+^ ATPase protein (Cy3 red **A**, **B**, **D**, **E**) was immunolocalized to the anterior rectum. Figures A and D contain overlays of Cy3 and Cy5 signals. (**B** and **E**) contain Cy3 signal only and Figures C and F contain Cy 5 only. Scale bars, 100·μm.

**Figure 2:**
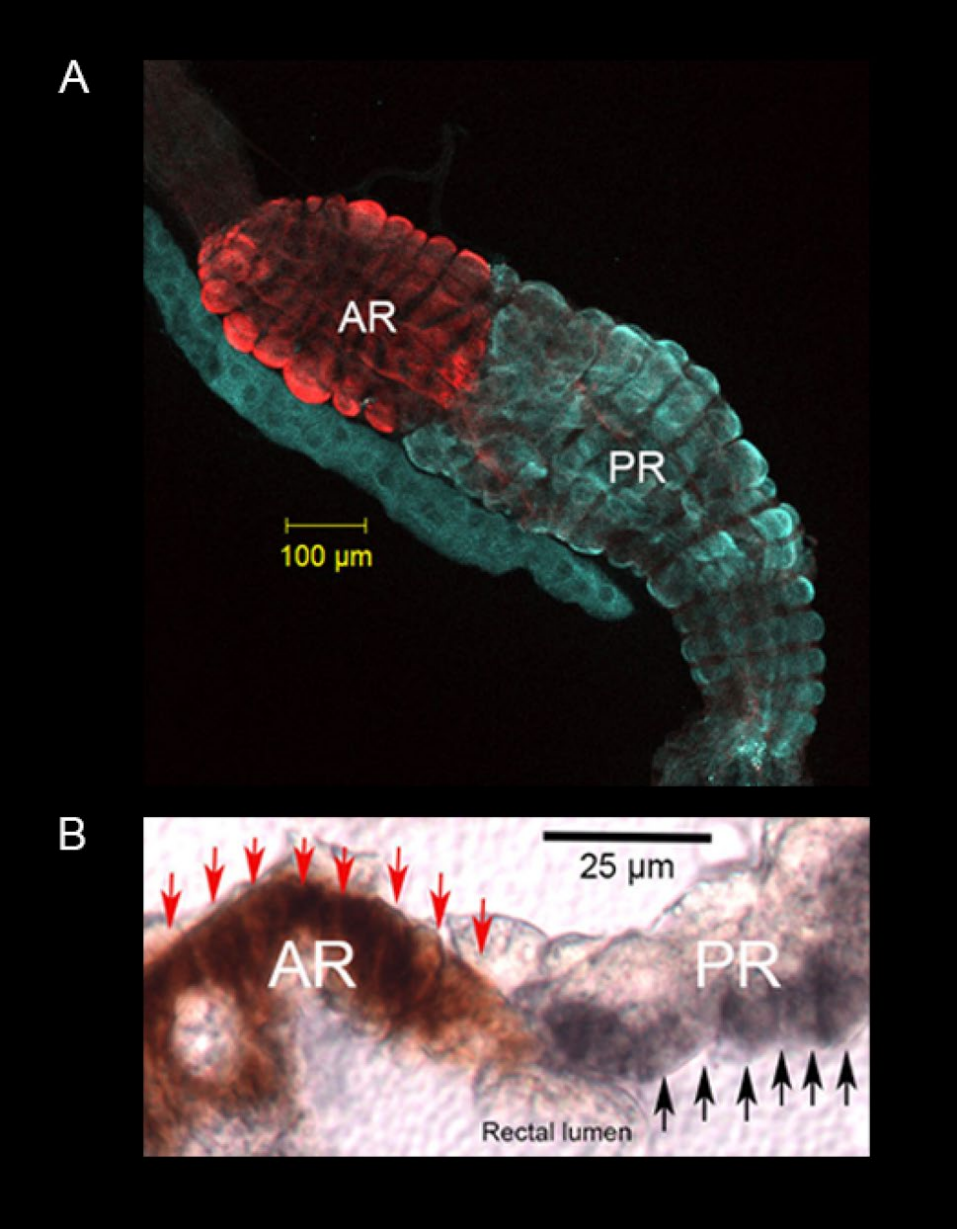
Expression of V-type H^+^ ATPase protein on the apical membrane of posterior rectal cells and P-type Na^+^/K^+^ ATPase protein expressed on basolateral membrane of anterior rectal cells of *A*.* taeniorhynchus* larvae. (**A**) Whole mount of the two-part rectum from larval *A*.* taeniorhynchus* acclimated to 100% seawater showing immunolocalization of V-type H^+^ ATPase protein (blue) to the posterior rectum (PR) and P-type Na^+^/K^+^ ATPase protein (red) to the anterior rectum (AR). (**B**) Sectional preparation of segmented rectum from a larva acclimated to 100% seawater indicating expression of the V-type H^+^ ATPase protein (dark grey staining, indicated by black arrows), on the apical membrane of PR facing the rectal lumen. P-type Na^+^/K^+^ ATPase protein (red staining, indicated by red arrows) was localized to the basolateral membrane of the AR that would face the hemocoele. Scale bars, 100 μm (**A**) and 25 μm (**B**).

A colony of *Aedes taeniorhynchus* was established in our University of California—Riverside and University of San Diego laboratories from colonies provided by USDA-Center for Medical, Agricultural and Veterinary Entomology Florida, USA. Mosquito larvae used in the propagation of the laboratory colonies were hatched and held in 1, 100 and 150% sea water using distilled water and Instant Ocean Salts (Aquarium Systems) in large rectangular plastic trays (32 cm × 18 cm × 9 cm) with the water changed every 4–5 days after reaching 3rd instar. Every 2 days, ~ 100–200 ml of ground liver and yeast slurry (made with distilled water) was added to each tray. Room temperature was 20–24 °C and the light:dark photoperiod was set at 12 h:12 h. Salinity was confirmed using a Wescor Vapor Pressure Osmometer (e.g., 100% seawater ~ 1000 mosmol kg^−1^). Experiments were conducted on fourth-instar larvae although, in some cases, large third-instar larvae may have been included. In one experiment (Figs. 1, 2), a group of larvae hatched and reared in 1% seawater were acutely transferred to 100% seawater. For scanning ion-selective electrode technique (SIET) measurements employing pharmacological agents (Fig. 7), *A*.* taeniorhynchus* eggs were supplied to our York University laboratory and hatched, reared and tested in 50–75% seawater. For SIET analyses of Na^+^ transport in FW and SW (Fig. 6), larvae were transferred to 1 and 100% seawater respectively for over 24 h.

### Immunohistochemistry and western blotting

For detailed methodology and antibody production for whole-mount and sectional immunofluorescence of V-type H^+^ATPase (VHA), P-type Na^+^/K^+^ ATPase (NKA) and Na^+^/H^+^ exchanger isoform 3 (NHE3) in mosquito larva recta, please refer to previous studies^24,25^. To localize P-type Na^+^/K^+^-ATPase protein, the monoclonal antibody, ‘a5’, raised against the alpha subunit of avian P-type Na^+^/K^+^-ATPase in mice by Dr Douglas Fambrough, was employed. This antibody was obtained from the Developmental Studies Hybridoma Bank (DSHB). This antibody was developed under the auspices of the National Institute of Child Health and Human Development and maintained by The University of Iowa, Department of Biological Sciences, Iowa City, IA 52242, USA. To localize V-type H^+^-ATPase, a polyclonal serum antibody raised against the B subunit of the V-type H^+^-ATPase of *Culex quinquefasciatus* was employed^26^. Whole serum and peptide-based NHE3 antibodies were generated in rabbit against the whole NHE3 protein or the C-terminal residues (E^1111^-G^1128^) respectively of *A*.* aegypti* NHE3^25^. Whole mount and tissue preparations were performed at the University of California, Riverside (CA, USA) and the University of San Diego (CA, USA). The two forms of NHE3 antibodies were used with section preparations of larval recta. Whole NHE3 antibody was diluted to 1:1000 and peptide NHE3 antibody was diluted to 1:250. Negative controls for polyclonal VHA and NHE3 were run with pre-immune rabbit serum (not shown). NKA negative control was run using DSHB supernatant (SP2/0). Whole mounts and tissue sections were examined using scanning confocal microscopy (Zeiss LSM510 Axioplan 2; located in core microscopy center at University of California, Riverside and San Diego). All images were imported into Adobe Photoshop for assembly and labelling.

For chromogenic detection of VHA and NKA in larval rectal sections, tissues were dissected, fixed and sectioned as described previously^24^. Vectastain Elite ABC avidin/biotin HRP chromogenic labelling system was used for immunodetection of both ATPases. First, sections were labelled using NKA primary antibody (1:6 dilution with Vectastain normal serum) and Vectastain NovaRed staining procedure. Next, sections were labelled using the primary antibody VHA (1:1000 dilution with Vectastain normal serum) and Vectastain DAB staining procedure. Sections were mounted using VectaMount medium, with coverslips on top and stored at 4 °C. Imaging of labelled ATPases in larval recta was performed using light microscopy with an Olympus BX51 compound microscope. All images were imported into Adobe Photoshop for assembly and labelling.

Western blotting of VHA and NHE3 in mosquito larval recta and Malpighian tubules, were carried out at the University of San Diego (CA, USA). Biological samples consisting of pools of larval posterior recta, anterior recta and Malpighian tubules that were isolated from 30 larvae under ice-cold saline were collected and stored at − 80 °C until later processing. For examination of expression, samples were thawed on ice, 300 μl of homogenization buffer (50 mmol l^−1^ Tris-HCl pH 7.4, 1 mmol l^−1^ PMSF and 1:200 protease inhibitor cocktail (Sigma-Aldrich)) was added to each 1.5 ml centrifuge tube containing tissue. Samples were homogenized on ice for 30 s using a hand-held tissue grinder (Kontes Pellet Pestle). Homogenates were then centrifuged at 10,000 g for 10 min at 4 °C and supernatant collected in a fresh tube and placed on ice. Supernatant protein content was then concentrated using Amicon Ultra cell 0.5 ultra-filtration centrifugal kit. Sample (300 μl) was loaded into each filter device and concentrated at 14,000 g for 40 min at 4 °C. Protein content of concentrated supernatant was determined using the Bradford assay (Bio-Rad) according to the manufacturer’s guidelines. Samples were prepared for SDS-PAGE by heating for 10 min at 95 °C in 5 × loading buffer containing 225 mmol l^−1^ Tris–HCl pH 6.8, 3.5% (w/v) SDS, 35% glycerol, 12.5% (v/v) β-mercaptoethanol and 0.01% (w/v) Bromophenol Blue. For VHA blotting, 25 μg of sample protein was loaded. For NHE3 blotting, 9, 12 and 38 μg protein for anterior recta, posterior recta and Malpighian tubule samples were loaded respectively. Sample proteins were electrophoretically separated by SDS-PAGE. Western blot analysis was carried out by overnight transfer of protein to Immobilon-P polyvinylidene-difluoride (PVDF) membrane (EMD Millipore, USA) at 4 °C followed by detection of VHA and NHE3 using Life Technologies Western Breeze anti-rabbit chemiluminescent kit and whole serum VHA and NHE3 antibodies diluted to 1:10,000 and 1:1000 respectively. Detection of protein bands was performed using Bio-Rad ChemiDoc XRS system.

### Gut lumen pH imaging

Six, fourth-instar larvae reared in 30 and 100% seawater were transferred to wells in a 24 well cell culture plate containing 1.5 ml filtered 30 and 100% seawater. Phenol red was dissolved in DMSO to make a 1% stock which was then aliquoted to each well to reach a final 0.1% value of the dye. Larvae were held in the phenol red media for one hour. One at a time, larvae were removed from the well, rinsed in fresh 30 or 100% seawater media, then transferred to a dissecting plate with PBS. The entire gut was carefully removed so it remained intact in order to avoid any disturbance of the gut contents, placed in a drop of PBS on a microslide, and imaged using an Olympus BX51 compound microscope. Images were imported into Adobe Photoshop for assembly and labelling.

### Scanning ion-selective electrode technique (SIET)

#### Pharmacological inhibitors

Bafilomycin (V-type H^+^-ATPase inhibitor, BioMol, Plymouth Meeting, PA, USA) was dissolved in dimethylsulfoxide (DMSO, Sigma Aldrich, Oakville, ON Canada), diluted in physiological saline to a concentration of 0.1 mmol l^−1^ and stored as aliquots of 40 μL at − 20 °C until needed. The physiological saline was adjusted to pH 7.0 and contained (in mmol l^−1^) 10 NaCl, 60 N-methyl-D-glucamine, 3 KCl, 5 NaHCO_3_, 0.6 MgSO_4_, 5 CaCl_2_, 25 HEPES, 5 L-Proline, 9.1 L-glutamine, 8.74 L-histidine, 14.4 L-leucine, 3.37 L-arginine HCl, 10 glucose, 5 succinic acid and 5 malic acid. An aliquot was added directly to a bath of 500 μL of physiological saline containing the preparation for a final bafilomycin concentration of 8 μmol l^−1^. The final test solution contained 0.8% DMSO. S3226 (NHE3 inhibitor, Sanofi-Aventis, Frankfurt, Germany) was dissolved in DMSO to a concentration of 10 mmol l^−1^ and stored at room temperature in the dark for no more than 7 days as recommended by the manufacturer. This stock solution was diluted in physiological saline to the final test concentration of 1 μmol l^−1^. The final test solution contained 0.01% DMSO. Bumetanide was dissolved in DMSO to a concentration of 10 mmol l^−1^ and stored at room temperature for no more than 1 day. This solution was diluted in physiological saline (described above) to a final test concentration of 10 µmol l^−1^.

#### Construction of ion-selective microelectrodes and SIET measurements of ion gradients adjacent the surface of the rectum

Liquid–membrane Na^+^ selective microelectrodes were manufactured as described previously^27^ using Na^+^ ionophore II cocktail A (Fluka, Buchs, Switzerland) and a back-fill solution of 100 mmol l^−1^ NaCl. The microelectrode tip diameters and ionophore cocktail column lengths were typically ~ 5 μm and 250–300 μm, respectively.

An assay for the SIET measurement of Na^+^ concentration gradients near the surface of mosquito larval rectums was developed. Fourth instar larvae were pinned to the bottom of a Sylgard™ (Dow Corning, Toronto, ON. Canada) coated cell culture dish that was filled with 5 mL of physiological saline (described above). A minuten pin was inserted through the thorax without puncturing the gut and the cuticle surrounding the rectum was removed. A second minuten pin was subsequently inserted through the cuticle that surrounds the anal opening at the base of the anal papillae to limit movements of the rectum. In this manner the tip of a microelectrode could be positioned within 5 μm from the surface of the rectum. This preparation was utilized to record and compare baseline Na^+^ fluxes from the rectums of larvae that were held in FW or 100%SW.

A second preparation was developed where the gut was cut at the intestine (anterior to the rectum) and at the anal canal (posterior to the rectum) and the rectum was placed on the bottom of a poly—L-lysine coated culture dish The culture dish contained a specifically manufactured steel insert with a trough allowing the total bathing volume to decrease to 500 μL. This preparation was utilized for the studies that examined the effects of bafilomycin and S3226 on the Na^+^ fluxes at the rectum. These studies consisted of recording the baseline Na^+^ fluxes from the rectum and re-recording the Na^+^ fluxes from the same sites along the same rectum 5 min after the addition of bafilomycin, S3226, bumetanide or DMSO (which served as the solvent control). All of the rectums employed in these studies were taken from larvae that had been reared in 50–75% seawater.

The SIET system and protocol employed in this study is described in detail previously^27^. Excursion distances of 100 µm were employed and the ‘wait’ and ‘sample’ periods were 4 and 1 s, respectively. Fluxes were recorded as an average of 5 repetitive measurements at each site. Na^+^ flux was calculated after subtracting the noise at a reference position 1–3 mm from the preparation from the differential signal measured at the site of interest near the preparation. The signal to noise ratios varied from a low of 5 to a high of 150. The Na^+^ microelectrodes were calibrated in 2.5, 25 and 250 mmol l^−1^ solutions of NaCl. To equalize the total ionic strength of the three solutions 225 and 247.5 mmol l^−1^ of LiCl was added to the 25 and 2.5 mmol l^−1^ solutions of NaCl, respectively. Microelectrode slopes (mV) for a tenfold change in ion concentration were (mean ± s.e.m, (N)): 51.4 ± 0.16, (27). Potential effects of inhibitors on the microelectrode were assessed by comparing the signals (in mV) in the calibration solutions with the signals in the same solutions containing 8 μmol l^−1^ bafilomycin, 1 μmol l^−1^ S3226 or 10 μmol l^−1^ bumetanide.

#### Calculation of ion fluxes

Voltage gradients were converted into concentration gradients using the following equation:

$$\Delta {\text{C }} = {\text{ C}}_{{\text{B}}} \cdot 10^{{(\Delta {\text{V}}/{\text{S}})}} {-}{\text{ C}}_{{\text{B}}}$$

where ΔC is the concentration gradient between the two points measured in μmol l^−1^ cm^−3^; C_B_ is the background ion concentration, calculated as the average of the concentration at each point measured in μmol l^−1^; ΔV is the voltage gradient obtained from ASET software in μV; and S is the slope of the electrode.

The concentration gradient was subsequently converted into flux using Fick’s law of diffusion in the following equation:

$${\text{J}}_{{\text{I}}} = {\text{ D}}_{{\text{I}}} (\Delta {\text{C}})/\Delta {\text{x}}$$

where J_I_ is the net flux of the ion in pmol cm^−2^ s^−1^; D_I_ is the diffusion coefficient of Na^+^ (1.55 × 10^–5^ cm^2^ s^−1^); ΔC is the concentration gradient in pmol cm^−3^; and Δx is the distance between the two points measured in cm.

#### Estimates of net Na^+^ transport at individual rectums

The fluxes at individual sites were averaged for the posterior rectum and the anterior rectum and multiplied by the surface area of the rectal segment to report the Na^+^ transport rates. The rectum of larval *A*.* taeniorhynchus* was divided into 2 distinct regions based on the morphology previously described in the introduction. The anterior and posterior rectum can be easily distinguished visually by the distinct morphology. An ocular micrometer was used to measure the widths and lengths of the anterior and posterior regions of 10 individual rectums. These measurements were used to calculate the relative surface areas of each region assuming a cylinder with open ends using the formula: SA = 2πr · *l*, where r and *l* represent the radius and length of the cylinder, respectively. The mean surface areas of the anterior and posterior regions calculated from the 10 rectums was multiplied by the calculated mean Na^+^ fluxes measured at each region to obtain the Na^+^ transport.

#### Statistics

Data are expressed as mean ± SEM (n). Na^+^ transport rates of the anterior and posterior segments of the rectum from FW and 100%SW larvae were compared using Welch’s t-test. A paired t-test was employed to examine the effects of inhibitors or DMSO (solvent control) by comparing the Na^+^ transport rates recorded prior to the addition of the inhibitor or DMSO with those recorded 5 min after the addition of the inhibitor or DMSO from the same rectum preparation. Baseline Na^+^ transport rates prior to the addition of inhibitor or DMSO were compared for each group of recta (e.g., those used to assess bafilomycin, S3226, bumetanide and DMSO, respectively) using a one-way ANOVA.

## Results

Whole mounts of recta from larval *A. taeniorynchus* held in 1% seawater and 24 h following an acute transfer from 1 to 100% seawater showed similar high expression levels of VHA in the posterior rectal segment and NKA in the anterior rectal segment (Fig. 1A–F). VHA expression in the posterior rectum appeared to be higher in the 100% seawater larvae (Fig. 1F) when compared to the 1% seawater sample (Fig. 1C). There were no or low detectable levels of NKA expression in the posterior recta under both salinity treatments (Fig. 1B,E). VHA expression in the anterior rectum appeared to be higher in the 1% seawater larvae (Fig. 1C) when compared to the 100% seawater sample (Fig. 1F) and NKA expression appeared similar (Fig. 1B,E). Negative controls for VHA (rabbit preimmune serum) and NKA (DSHB SP2/0 conditioned medium labelling in tissue whole mounts resulted in very low or no signal (not shown).

The whole mount of recta from larval *A. taeniorynchus* 24 h following an acute transfer to 100% seawater from 1% seawater showed high expression of VHA in the posterior rectal segment and similar high expression of NKA localized to the anterior rectal segment (Fig. 2A, same as Fig. 1D). In paraffin sections of the anterior and posterior recta of larval *A. taeniorynchus* reared in 100% seawater, VHA was immunolocalized to the apical membrane of the posterior rectal cells and NKA was localized to the basal membrane of the anterior rectal cells (Fig. 2B). There were no or low detectable levels of expression of NKA in the posterior rectal cells and VHA in the anterior rectal cells.

**Figure 3:**
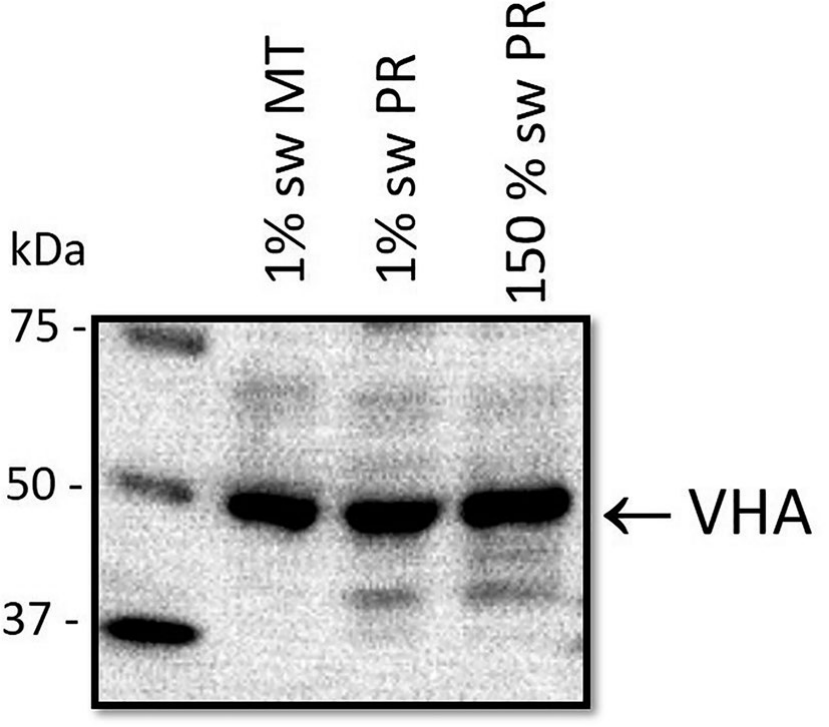
Expression of V-type H^+^ ATPase protein in *A*.* taeniorhynchus* larvae. Western blot analysis of (VHA) protein with molecular mass standards (left lane) and homogenate of Malpighian tubules (second lane) and posterior recta (third lane) from 1% seawater held larvae and posterior recta (fourth lane) from 150% seawater held larvae revealing a band of ∼ 50 kDa.

Western blot probed with VHA B subunit antibody of posterior rectal tissue homogenate from larval *A. taeniorynchus* held in 1% and 150% seawater revealed a single band at ~ 50 kDa band (Fig. 3). 1% seawater Malpighian tubules had similar level of VHA expression and served as a positive control for VHA detection.

**Figure 4:**
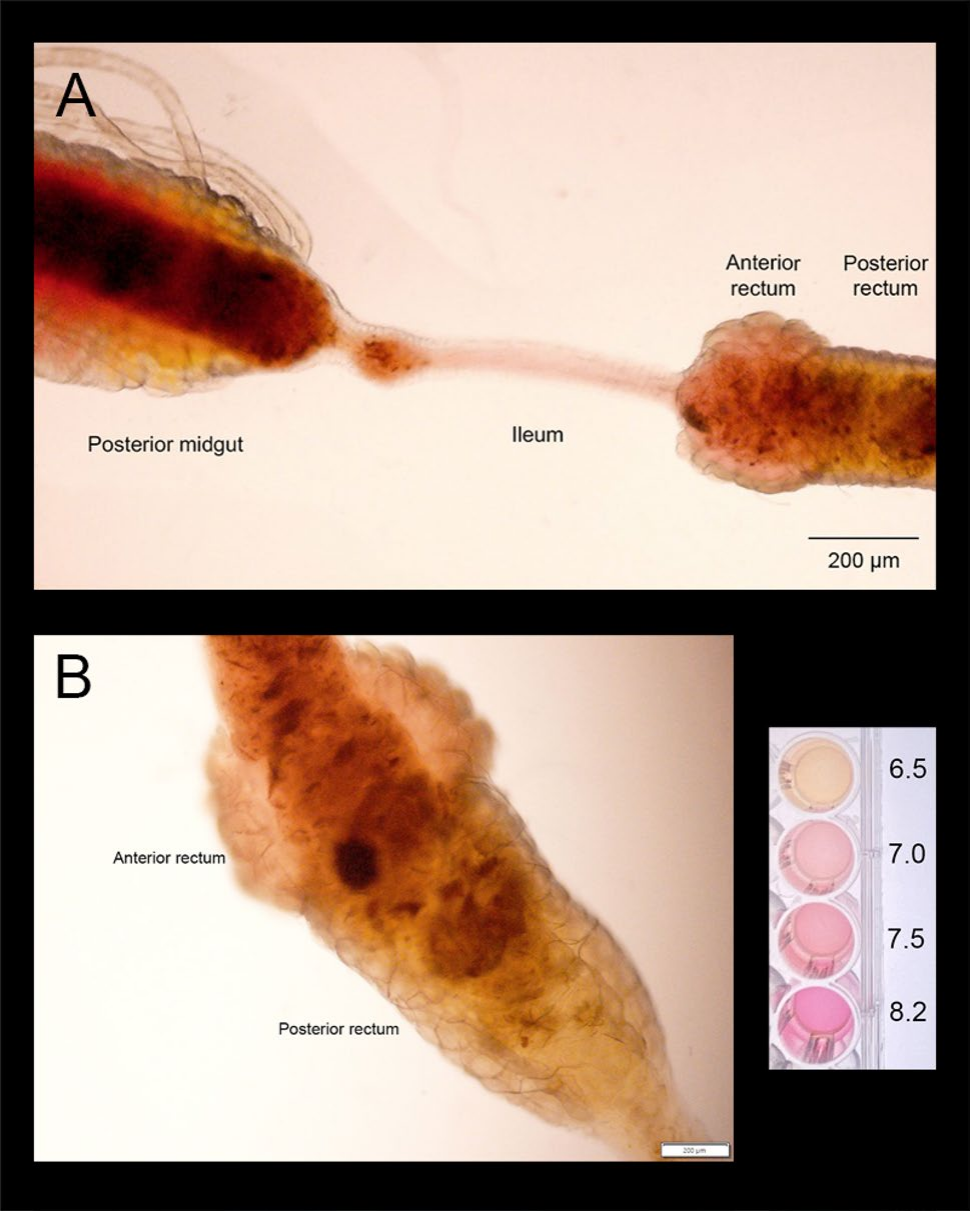
Hindgut lumen pH visualized with phenol red indicator dye of *A*.* taeniorhynchus* larvae reared in 100% seawater. Intact alimentary canal was dissected from larvae following one hour incubation ion 0.1% phenol red in 100% seawater medium. Scale of pH colour with Phenol Red diluted in 100% seawater titrated to pH 6.5, 7.0, 7.5 and untitrated seawater with a pH of 8.2.

Gut lumen pH of *A. taeniorynchus* held in 100% seawater was imaged using Phenol Red indicator dye (Fig. 4). A yellow colour indicating a more acidic urine (≤ pH 6.5) was evident in the posterior rectal lumen whereas a light pink fluid, indicating a more alkaline urine (≥ pH 7), was observed in the anterior rectal lumen (Fig. 4A,B). This trend was also evident in the hindgut of larvae reared at 30% seawater (not shown). The drop in pH from anterior to posterior segments of the rectum is similar to that noted in the anterior to posterior segments of the midgut; however, colour intensity is far greater in midgut (Fig. 4A). It is also noted in Fig. 4A that fluid in the ileum (or anterior intestine) was also light pink similar to the anterior rectum.

**Figure 5:**
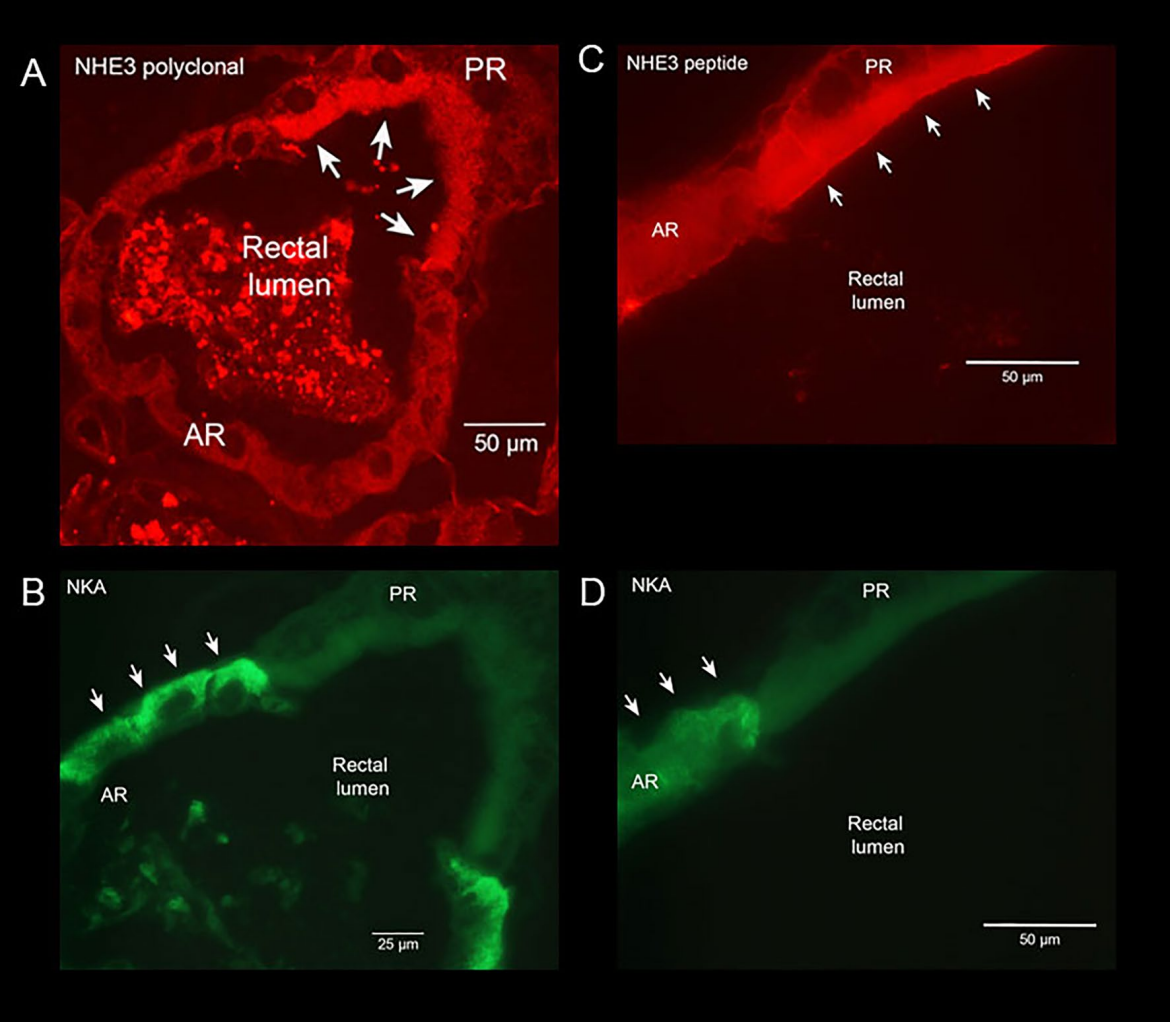
Expression of NHE3 protein on the apical membrane of posterior rectal cells and P-type Na^+^/K^+^ ATPase protein expressed on basolateral membrane of anterior rectal cells of *A*.* taeniorhynchus* larvae. Sectional preparation of segmented rectum of larval *A*.* taeniorhynchus* acclimated to 100% seawater showing immunolocalization of NHE3 protein (red) using NHE3 polyclonal antibody (**A**) and NHE3 peptide (**C**), on the apical membrane of PR facing the rectal lumen (indicated by white arrows). P-type Na^+^/K^+^ ATPase protein (green) was immunolocalized to the basolateral membrane of the AR (indicated by white arrows) that would face the hemocoele. (**B**, **D**). Scale bars, 50 μm (**A**, **C**, **D**) and 25 μm (**B**).

Paraffin sections of recta of *A. taeniorynchus* larvae reared in 100% seawater showed high levels of NHE3 expression on the apical membrane of posterior rectal cells (Fig. 5A,C). Both the polyclonal, serum-based NHE3 antibody (Fig. 5A) and the peptide-based NHE3 antibody (Fig. 5B) showed high levels of NHE3 expression in the posterior rectal cells and no or low detection levels in the anterior rectal cells. The basal membrane of anterior rectal cells was identified by the high NKA expression levels compared to the low or no detection of NKA in the posterior rectal cells (Fig. 5B,D). Negative controls for NKA (DSHB SP2/0 conditioned medium) and NHE3 (rabbit preimmune serum) labelling in tissue sections resulted in very low or no signal (not shown).

**Figure 6:**
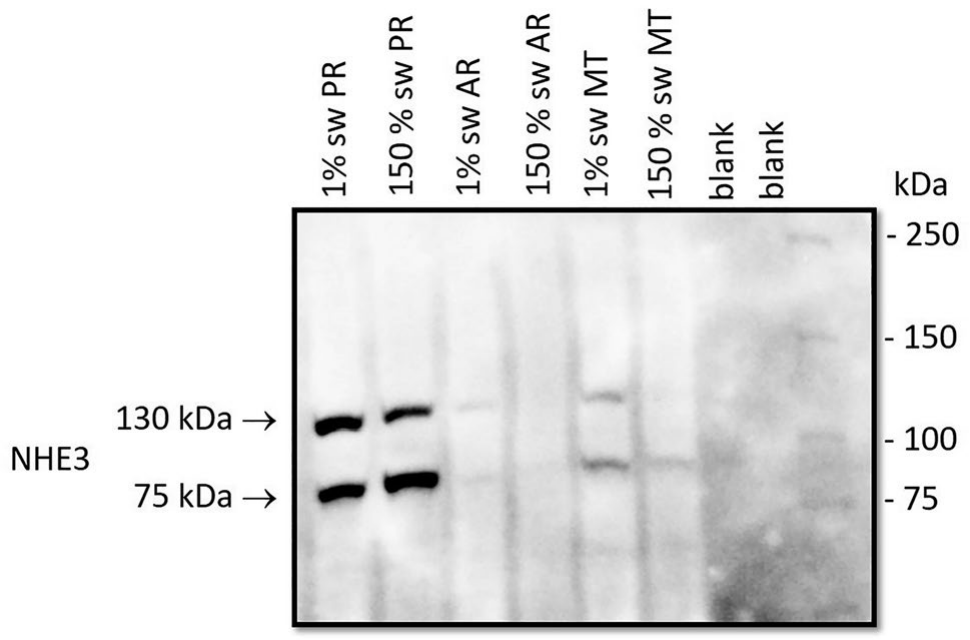
Expression of Na^+^/H^+^ exchanger isoform 3 (NHE3) protein in *A*.* taeniorhynchus* larvae. Western blot analysis of (NHE3) protein with molecular mass standards (right lane) comparing homogenates of posterior recta (first and second lane), anterior recta (third and fourth lane) and Malpighian tubules (fifth and sixth lane) from *A*.* taeniorhynchus* larvae acclimated to 1% and 150% seawater. Two bands were detected at 75 and 130 kDa in each tissue. The polyclonal NHE3 antibody was used.

Western blot probed with polyclonal NHE3 antibody of posterior rectal, anterior rectal and Malpighian tubule tissue homogenate from larval *A. taeniorynchus* held in 1% and 150% seawater revealed two bands at ~ 130 and 75 kDa (Fig. 6). The posterior rectal homogenates expressed both bands with the 150% seawater sample… All other tissue samples from 1 and 150% seawater larvae expressed both NHE3 bands except for the 150% seawater Malpighian tubule sample where only the 75 kDa band was detected. The Malpighian tubles from 1% seawater reared larvae served as a positive control for NHE3 detection.

**Figure 7:**
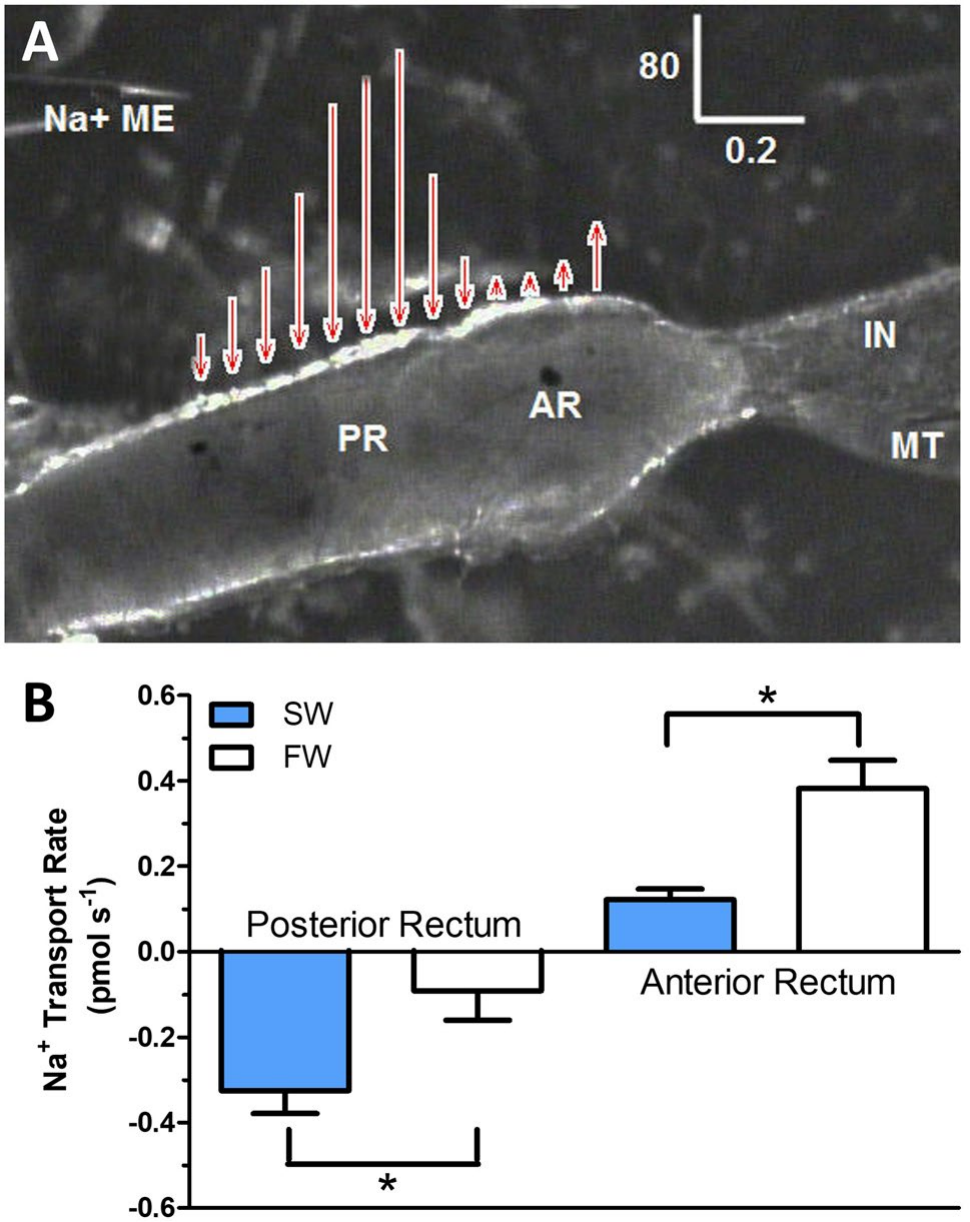
Na^+^ transport by the rectum of *A*.* taeniorhynchus* larvae. (**A**) A representative scan of Na^+^ flux at locations along the rectum of larval *A*.* taeniorhynchus*. The length and direction of the arrows represent the magnitude and direction of net Na^+^ transepithelial flux respectively (IN = intestine; MT = Malpighian tubule; Na^+^ ME = Na^+^ selective microelectrode). Scale bar units are in pmol cm^−2^ s^−1^ (vertical = flux) and mm (horizontal). (**B**) The rate of Na^+^ transport at the posterior rectum and the anterior rectum of *A*.* taeniorhynchus* larvae reared in freshwater (FW) or seawater (SW). A positive rate of transport indicates efflux (from lumen to bath) and a negative rate of transport represents influx (from bath to lumen). Values are expressed as mean ± SEM with n = 6 for FW and n = 12 for SW. A comparison of Na^+^ transport rates in FW and SW larvae was performed with unpaired t-test for both posterior (*p* = 0.02) and anterior rectum (*p* < 0.001).

In situ, sodium fluxes across intact recta of larval *A. taeniorynchus* held in FW or 100% seawater were measured using SIET (Fig. 7). Localized Na^+^ flux was illustrated by vectors superimposed on a digital image of the two-part rectum (Fig. 7A). The direction of the vector reflects the movement of Na^+^ into (secretion) or out of (absorption) the rectal lumen, whereas the length of the vector reflects the magnitude of the Na^+^ flux (Fig. 7A). Posterior recta from seawater-held larvae exhibited a significantly higher rate of Na^+^ secretion, approximately a threefold greater rate compared to freshwater-held posterior recta (Fig. 7B). Anterior recta from seawater-held larvae had a significantly lower Na^+^ absorption rate, approximately one fourth that of freshwater-reared larval anterior recta (Fig. 7B).

**Figure 8:**
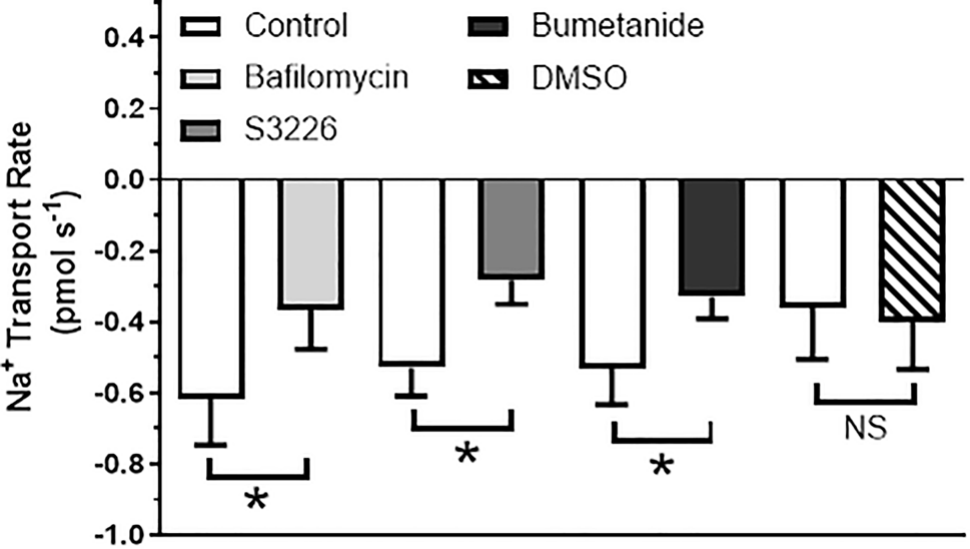
Inhibition of Na^+^ transport at the posterior rectum by V-type H^+^-ATPase, NHE3 and NKCC inhibitors. Na^+^ transport rate was measured at the posterior rectum of *A*.* taeniorhynchus* larvae that were reared in seawater before and after application of bafilomycin A1 (V-type H^+^-ATPase inhibitor, 8 μmol l^−1^), S3226 (NHE3 inhibitor, 1 μmol l^−1^), bumetanide (NKCC inhibitor, 10 µM) or dimethylsulfoxide (DMSO, inhibitor solvent, 0.8%). Values are expressed as mean ± SEM with n = 9 for bafilomycin, n = 11 for S3226, n = 9 for bumetanide and n = 7 for DMSO. Effects of each treatment were assessed by comparing Na^+^ transport rates before (Control) and after inhibitor or solvent with a paired t-test (*p* = 0.024 for bafilomycin; *p* = 0.025 for S3226; *p* = 0.017 for bumetanide; *p* = 0.754 for DMSO). Na^+^ transport rates of before inhibitor/solvent did not statistically differ from one another (ANOVA, *p* = 0.502).

In situ, sodium fluxes measured across intact posterior recta of larval *A. taeniorynchus* held in diluted seawater (50–75% seawater) showed sensitivity to VHA, NHE3 and NKCC inhibitors (Fig. 8). Na^+^ secretion into the posterior rectal lumen was significantly reduced by ~ 30% in the presence Bafilomycin A_1_, a VHA inhibitor, ~ 50% in the presence of S3226, a NHE3-specific inhibitor, and ~ 40% in the presence of bumetanide, a NKCC inhibitor in comparison to control flux measurements. DMSO, the solvent used with both inhibitors, did not have a significant effect on Na^+^ secretion rates (Fig. 8).

## Discussion

**Figure 9:**
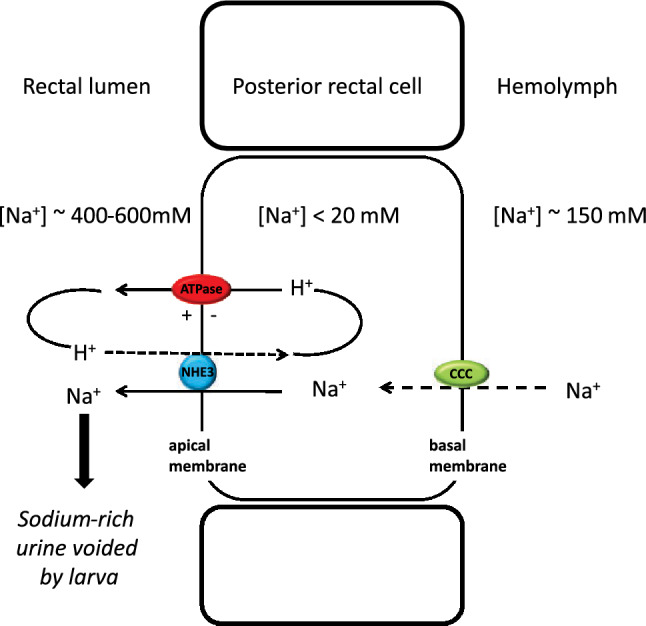
Model of Na^+^ secretion mechanism in the posterior rectum of larval *A*.* taeniorhynchus* held in high salinity water. A cation-chloride co-transporter**,** CCC, transporter is the site of Na^+^ entry into the cell across the basolateral membrane. The apical V-type H^+^-ATPase (VHA) pumps protons from the posterior rectal cell into the rectal lumen to establish an electromotive gradient (inside negative). Lumenal protons diffuse down this inward gradient through the apical Na^+^/H^+^ exchanger, NHE3, to drive the secretion of Na^+^ via this exchanger against the large Na^+^ gradient. This H^+^- driven Na^+^ secretion generates the hyperosmotic, Na^+^-rich urine^10^.

We have characterized a novel H^+^ driven-Na^+^ secretion mechanism that is key to the ionoregulatory strategy of the strongly euryhaline *Aedes taeniorhynchus* larva. SIET analyses revealed differential regulation of Na^+^ fluxes in the anterior (influx reduced) and posterior (secretion increased) recta to realize an overall net Na^+^ secretion into the urine in larvae held in high salinity (Fig. 7B). Our study provides protein expression and functional evidence of the proton pump, V-type H^+^ ATPase, driving Na^+^ secretion via a Na^+^/H^+^ exchanger (NHE3) across the apical membrane of posterior rectal cells. This is the primary route to excrete excess Na^+^ that larvae ingest when drinking the external salty water as part of their fluid homeostasis strategy. We also determined that Na^+^ secretion across the posterior rectum is bumetanide-sensitive (Fig. 8) suggesting a cation-chloride cotransporter (CCC) operating on the basal membrane. Our findings build upon previous studies that documented powerful ion transport function in the posterior segment that is capable of yielding urine 2–3 times more concentrated than hemolymph values^20^. This system departs from all other documented strategies in salt-tolerant animals, both vertebrate and invertebrate, where the sodium pump (i.e. Na^+^/K^+^ ATPase) is employed to power the elimination of excess sodium^21,22^. Our model (Fig. 9) also differs from the urine concentrating strategies of terrestrial insect species where local osmotic gradients, generated across elaborated lateral membrane systems of rectal cells, drive water reabsorption from urine (reviewed by O’Donnell, 2022^22^). The primary ATPase powering this terrestrial water reabsorption system remains to be identified as well as in other noted salt-tolerant aquatic insects (*Ephydrella*^28^), including anopheline mosquito larvae^29^.

Our protein expression analyses of the two-part rectum of *A*.* taeniorhynchus* indicate that VHA is the sole ATPase housed in the posterior rectal cells (Figs. 1, 2), is abundantly expressed, and localized to the highly folded apical membrane (Fig. 2b) in larvae held in both low and high salinity (Figs. 1, 3). VHA immunoblots (Fig. 3) clearly show a single band corresponding to the predicted size of approximately 50 kDa for the VHA B subunit protein^26^. Our findings also align with previous transmission electron microscopy studies that described a particulate coating of the highly elaborate posterior rectal apical membrane of *A*.* campestris* and anal canal of *A*.* togoi*, both salt-tolerant, culicine species^16,18^. These particulates, also known as portasomes, have been noted in a diversity of insect ion transport epithelia^30^, mainly on the cytoplasmic aspects of apical foldings, and have been confirmed to be the V_1_ complex of VHA^31^. A previous study^29^, employing the same VHA/NKA immunolocalization protocol on whole mounts of *A*.* taeniorhynchus* recta as we had first reported^32^, confirms our pattern of VHA expression in the posterior rectum in this current study (Figs. 1, 2).

Having demonstrated the expression and localization of VHA in the posterior rectum, we provided functional evidence of VHA by visualizing the pH decrease as urine moves from the anterior to the posterior segment of the rectum of *A*.* taeniorhynchus* larvae held in and ingesting 100% seawater (Fig. 4). The urine held in the anterior segment is more alkaline (pH of 7 or higher) when compared to the urine residing in the posterior segment that was yellow in colour, indicating a pH of 6.5 or lower (Fig. 4A,B). Another study examined larval gut pH in several mosquito species, including *Ochlerotatus taeniorhynchus* (former species name), held in freshwater. In all other species examined, hindgut fluid pH to appear neutral but unfortunately, there was no hindgut fluid or colour in the rectum of this salt-water species^33^. Assuming that the intracellular pH of the posterior rectal cell is approximately 7.0–7.5 (based on intracellular pH measurements of *A*.* aegypti* larval posterior midgut cell^34^), and the ingested seawater has a high buffering capacity, together, indicates that a substantial amount of protons are pumped into the lumen of the posterior segment to generate a favourable pH gradient needed to drive Na^+^ secretion via the electroneutral exchanger, NHE3.

We used SIET analyses to demonstrate the intimate coupling between H^+^ pumping and Na^+^ secretion in a saline-water animal. Our direct measurement of a significant inhibition of Na^+^ flux by VHA antagonist, bafilomycin A_1_, provided functional evidence of VHA powering Na^+^ secretion across the posterior rectal epithelium (Fig. 8). Additional SIET work showed higher Na^+^ secretion rates across the posterior rectal segment in larvae held in high salinity compared to larvae residing in lower salinity (Fig. 7). This trend parallels Bradley and Phillips^9,10^ report of greater Na^+^ concentration in rectal fluid collected from *A*.* taeniorhynchus* larvae held in 100 and 200% seawater compared to 10% seawater and the greater TEP and Na^+^ secretion rates when in vitro preparations of posterior recta were exposed to artificial hemolymph with elevated Na^+^ levels (200 mM).

The next question to ask is how a VHA-driven Na^+^ secretory mechanism is regulated in response to elevated salinity and/or hemolymph Na^+^ levels? Although qualitative, our protein blots may indicate a greater VHA protein density in posterior recta from larvae held in 150% seawater (24 h post transfer from 30% seawater) versus 1% seawater- rearing conditions (Fig. 3). Future quantitative assessment of VHA gene and protein expression will determine if transcriptional regulation of VHA underlies acute or long-term activity. However, there are few, if any, studies showing this mode of regulation in animals. Instead, salt stress induction of H^+^ ATPase expression has only been documented in salt-tolerant plants where this stress induces V-type and P-type H^+^ ATPase expression in key tissues involved in sequestering or eliminating excess Na^+^^35^. Forms of post-translational regulation of VHA function have been noted in animals that involve the association or dissociation of V_0_ and V_1_ complexes as means to stimulate or inhibit activity respectively following an acute challenge^36,37^. For example, serotonin regulates VHA assembly leading to enhanced activity as described in *Calliphora* salivary glands^38^. Kinin diuretic peptides applied to Malpighian tubules of adult *Aedes aegypti* are associated with the assembly of VHA complexes^39^. Bradley and Phillips^10^, however, found that application of serotonin, cAMP, and theophylline to in vitro preparations of *A*.* taeniorhynchus* posterior recta did not impact trans-rectal potential difference or secretion rate. More recently, using phosphoproteomics, Kandel et al.^37^ showed that the degree of VHA subunit phosphorylation in the Malpighian tubules of adult female *Aedes aegypti* did not differ between unfed and 1 h post blood meal when VHA activity is high and driving rapid urine production. Instead, VHA subunits are dephosphorylated 24 h post blood meal paralleling the reduction in ion and water transport in this organ. Given the rapid changes in P.D. and Na^+^ secretion by the posterior rectal segment of larval *A*.* taeniorhynchus*^10^ there must be other signaling systems that can quickly upregulate VHA activity, possibly through regulating the assembly of the subunits.

The extremely low NKA expression in the posterior rectal segment (Figs. 1, 2, 5B,D) is supported by the ouabain-insensitive trans-rectal potential difference and the lack of change in the basal membrane potential when trans-rectal potential difference was stimulated with high Na^+^ artificial hemolymph^10^. Smith et al.^29^ also reported low or no NKA expression in *A*.* taeniorhynchus* posterior recta. Despite the lack of functional evidence, Bradley and Phillips^10^ still included NKA on the basal membrane in their model but noted that basal NKA must be a “low capacity” system masked by the “high capacity electrogenic pumps” on the apical membrane which we have shown to be VHA (Figs. 1–3, 8). There are other examples of Na^+^ secreting, VHA-only cells, such as the ion-transporting cells in the gastric caeca organ of freshwater reared larval *Aedes aegypti*^40^, that were characterized using our current approach of protein expression and SIET analyses of Na^+^ flux, and the principal cells of the distal region of Malpighian tubules of blood-fed adult females^41^ and larval mosquitoes^39^, including *A*.* taeniorhynchus*^32^. In these cases, apically localized VHA is driving the secretion of Na^+^ and/or K^+^ into the lumen of these two organs to increase lumenal fluid osmotic concentration in the gastric caeca and to move water from the hemolymph into the Malpighian tubule lumen to generate primary urine.

In contrast, the anterior rectum expresses both VHA and NKA on the apical and basal membrane respectively (Figs. 1, 2) and exhibits Na^+^ absorption, as measured by SIET, with rates significantly higher when *A*.* taeniorhynchus* larvae are held in fresh water (Fig. 7). Our results, in conjunction with ultrastructural studies reporting a highly elaborate basolateral membrane system (“labyrinth”) and a moderately folded apical membrane^16^ aligns with an ion resorptive function as characterized by Bradley and Phillips^10^ and reminiscent of the structure and transport properties of the single rectum of fresh water larvae *A*.* aegypti*^6,16^. Smith et al.^29^ reported similar NKA/VHA expression patterns in their *A*.* taeniorhynchus* anterior rectal preparation.

Our combined protein expression and SIET functional analyses indicate that NHE3 is the Na^+^ transporter housed on the apical membrane of the posterior rectal segment that utilizes the energy of the H^+^ electromotive gradient generated by the apical VHA to secrete Na^+^. We detected high expression levels of two variants (75 and 130 kDa) of NHE3 protein in the posterior rectum of larval *A*.* taeniorhynchus* (Fig. 6), with both variants localized to the apical membrane using both a NHE3 polyclonal antibody (Fig. 5A) and a peptide antibody that recognizes the carboxy tail of NHE3 (Fig. 5C). Previous studies by Hart et al.^42^ and Pullikuth et al.^25^ had identified the presence of a splice site in the NHE3 gene of *A*.* aegypti* with predicted protein molecular weights approximate to what we documented for *A*.* taeniorhynchus* (Fig. 6). Interestingly, Pullikuth et al.^25^, using the same peptide NHE3 antibody as in this present study, reported that NHE3 was primarily localized to the basal membrane of several osmoregulatory organs of freshwater *A*.* aegypti* including the single segment rectum. However, it was found on the apical membrane of the median segment of the Malpighian tubules. Given the assumption that the anterior rectum of *A*.* taeniorhynchus* is homologous to *A*.* aegypti* rectum in structure and function^20^, we would predict NHE3 expression in the former when probed with both antibodies. However, our results indicate low NHE3 expression in the anterior rectum (Figs. 5A,C, 6). This disparity may be attributed to Pullikuth et al.^25^ employing only the peptide antibody which recognizes the 130 kDa NHE3 protein and not the truncated 75 kDa NHE3 protein. Conversely, the trends may suggest that there exist species and/or salinity tolerance differences in ion transporter populations in the freshwater and anterior rectum. Our localization of NHE3 to the apical membrane of the posterior rectal tissue parallels trends described in the brush borders of vertebrate renal^43^ and intestinal systems^44^. NHE3 splice variant expression and function, however, have yet to be characterized in these or other vertebrate and invertebrate species with the exception of *A*.* aegypti* mosquito^25^.

SIET analyses provided evidence of NHE3 functioning to secrete Na^+^ across the posterior rectal epithelium (Fig. 8). Application of a NHE3-specific pharmacological antagonist, S3226, resulted in a 50% inhibition of Na^+^ secretion rates (Fig. 8). S3226 was reported as a novel NHE3- inhibitor with high selectivity in mouse and opossum cells lines^45^ and has also been employed to confirm NHE3 function in ammonia excretion in *A*.* aegypti* anal papillae^46^ In contrast, amiloride analogs inhibit various Na^+^ transporters, including NHE isoforms and epithelial Na^+^ channels (eNaC), but exhibit low specificity for NHE3 isoform in several ion transporting systems^45,47^. Pullikuth et al.^25^ identified a mutation that causes a single amino acid substitution in the amiloride-binding domain in *Ae*NHE3 that explains amiloride-insensitive Na^+^ transport when this form is expressed in a fibroblast cell line. The same mutation was noted in *Drosophila* and mammal NHE3 but not crab NHE^25^. Initial SIET work using amiloride did not inhibit Na^+^ secretion in our posterior rectal preparation (not shown) suggesting that *At*NHE3 shares the same amiloride-binding domain mutation and hence amiloride insensitivity as *Ae*NHE3.

The presence of two NHE3 splice variants in the posterior rectum of larval *A*.* taeniorhynchus* (Fig. 6) raises the possibility of differential expression regulation by salt stress if one variant underlies the greater Na^+^ transport rate as documented to occur rapidly when bathing medium or hemolymph Na^+^ levels are acutely elevated^10^. Examination of *Ae*NHE3 putative protein sequence indicates several possible phosphorylation sites populating the elongated carboxy tail^25^. This same study reported that the heterologous expression of the truncated *Ae*NHE3 exhibited similar Na^+^ transport function as the full-length variant. From this, we can assume that the full length *At*NHE3 protein is regulated through several kinase signaling pathways whereas the truncated variant is freed from these modes of regulatory input and presumably could be functioning at a high level of activity without restriction. Support for the truncated NHE3 playing a bigger role in larvae held at high salinity comes from previous findings that reported elevated Na^+^ secretion and trans-rectal potential difference of in vitro preparations of *A*.* taeniorhynchus* posterior recta that were resistant to serotonin, cAMP, and theophylline^10^. Interestingly, distinct splice variants of NHEs have been documented in in *Anopheles*, *Drosophila* and *Caenorhabditis elegans*, including NHE3, with possible modes of regulating expression and function via carboxy tail sites extensively reviewed^25,48^. These variants might serve specific roles that are performed by NHE isoforms found in vertebrate systems. Key phosphorylation sites noted in the *Ae*NHE3 carboxy tail have been implicated in regulating the number of exchangers present (increase or decrease) on the membrane as found in vertebrate NHEs^48^ This could be the mechanism to realize higher Na^+^ secretion rates in the posterior recta of larvae held in higher salinity (Fig. 6). Future studies aim to quantify splice variant density in the posterior recta when *A*.* taeniorhynchus* larvae are reared inlow salinity then acutely transferred to high salinity.

Na^+^ transport across the posterior rectal segment was determined to be bumetanide sensitive (Fig. 8). This result indicates the presence of cation-chloride co-transporter that presumably serves as the Na^+^ entry step on the basolateral membrane (Fig. 9). Bumetanide is considered to be selective for the Na^+^ K^+^ 2Cl^−^ cotransporter isoform1^49^, however, we did not detect high levels of expression of NKCC1 protein in the *A*.* taeniorhynchus* hindgut tissue (data not shown) using an *A*.* aegypti* NKCC1 antibody^50^. Similarly, Filipov et al.^50^ reported high expression of NKCC1 in the midgut of larvae *A*.* aegypti* but little or no gene and protein expression in the hindgut of this freshwater-obligate species. In addition to NKCC1, a few other forms of cation-chloride co-transporters have been identified in *A*.* aegypti* including NKCC2, CCC2 and CCC3, with the latter expressed to higher levels in larval versus adult tissues but bumetanide sensitivity, based on amino acid sequence, is predicted to be less (NKCC2) or undetermined (CCCs)^49^. NKCCs have been characterized in other insect osmoregulatory epithelia^51^and serves as a key transporter in the NaCl secretion model for marine teleost ionocytes^52^. NKCCs in the Malpighian tubules of *Rhodnius prolixus* have been documented to function on the basal membrane^53^ and, following a blood meal by the insect, when hemolymph Na^+^ levels are elevated, excess Na^+^ competes for K^+^ transport in this transporter thus elevating the rate of Na^+^ secretion and turning NKCC into a 2Na:2Cl transporter. Future studies will identify the specific form of CCC involved in the basal membrane transport step for the novel Na^+^ secretory process in the posterior rectum of larval *A*.* taeniorhynchus* (Fig. 9).

Our current study presents both protein (Figs. 1–3, 5, and 6) and functional (Figs. 4, 7, and 8) evidence of the transporters responsible for the H^+^- driven Na^+^ secretion in saline water mosquito species and enables us to propose a model (Fig. 9). Given that hypo-osmoregulatory salinity tolerance is believed to have evolved several times independently within the *Culicidae* family^20^, we predict that this mechanism evolved concurrently with specialized hindgut epithelia, such as the posterior rectal segment and anal canal, within the culicine mosquitoes (*Ochlerotatus*, *Finlaya* subgenera). Interestingly, in anopheline subfamily, larvae of both fresh and saline water species possess recta consisting of two cell types that are regionalized into the dorsal anterior region (DARS) and the main portion of the rectum (non-DAR)^54^. A previous study had characterized *An*.* ainshamsi* larvae as saline tolerant hypo-osmoregulators but had not reported if the rectal organ in this anopheline species possessed a specialized epithelia and could generate hyperosmotic urine as described in saline water *Aedes* species^12^. Recent studies^29,55^ reported that the non-DAR cells possess a highly folded apical membrane, expresses VHA and exhibits a bafilomycin-sensitive H^+^ transport into the rectal lumen. However, their results also show that freshwater (*An*.* gambiae*) and saline water species (*An*.* albimanus*) have similar NKA and VHA expression patterns in both cell types in both fresh and brackish rearing conditions, the non-DAR apical membrane extends only 30% into the cell versus 60% in *A*.* campestris* posterior rectum^16^ or *A*.* togoi* anal canal, and there was no difference in H^+^ flux rates as measured using SIET in larvae reared in different salinities. Their combined SIET and immunolocalization results suggest that VHA is responsible for the proton fluxes across both cell types but doesn’t show a differentiation in data trends to support the hypothesis that a VHA-driven Na^+^ secretion process is occurring in the non-DARs segment. Instead, the greater NKA expression in DAR cells in larval *An*.* albimanus* versus *An*.* gambiae* when held in high salinity could imply an NKA-driven Na^+^ secretion. These findings are highly intriguing as they may show diversity in how hyperosmotic urine is generated in the mosquito family where a diversity of specialized hindgut organs have evolved in saline water species.

On a broader, evolutionary scale, this H^+^- driven Na^+^ secretion mechanism may reflect freshwater versus seawater origin in the ATPase selection to drive ionoregulation in insect versus vertebrate and invertebrate aquatic groups. For example, marine teleosts employ NKA to drive NaCl secretion in gill ionocytes to hypo-ionoregulate^52^. Marine invertebrates have evolved to ionoconform or weakly hypo- osmoregulate in seawater and rely upon NKA in their osmoregulatory tissues to aid in maintaining water balance and ammonia excretion^51^. In contrast, terrestrial insects that evolved an aquatic life stage did so by exploiting freshwater habitats^56^. For example, evidence of a freshwater ancestry for the mosquito Family *Culicidae* stems from the documentation of ~ 95% of all extant species being freshwater obligate and their closest relatives, Families *Dixidae* and *Chaoboridae*, being strictly freshwater^57,58^. There are many examples of insect groups possessing aquatic stages with the ability to tolerate highly saline water^56^ but few have had the ion transport mechanism characterized to the same depth as saline water mosquitoes. More examples of H^+^- driven Na^+^ secretion may be discovered in other insect groups possessing salt-tolerant life stages (e.g. *Ephydrella*^28^) or are capable of generating hyperosmotic urine via ion secretion (e.g. *Sarcophaga*^59^). Such adaptations may help explain the success of salt-tolerant insects to rapidly expand their larval niches and exploit highly productive coastal or inland habitats with high salts and/or unusual chemistry (high HCO_3_, SO_4_^–2^)^7^ as Na^+^ regulation is not reliant upon the maintenance of an NKA-driven Na^+^ electrochemical gradient.

## Data Availability

The datasets generated and analyzed during the current study are available from the corresponding author upon reasonable request.
