# Author Correction: Proton-driven sodium secretion in a saline water animal

**DOI:** 10.1038/s41598-024-74220-y

**Published:** 2024-10-10

**Authors:** Marjorie L. Patrick, Andrew Donini, Andrew Zogby, Christopher Morales, Michael J. O’Donnell, Sarjeet S. Gill

**Affiliations:** 1 Department of Biology, University of San Diego, 5998 Alcalá Park, San Diego, CA 92111 USA; 2 Department of Biology, York University, 4700 Keele St, Toronto, ON M3J 1P3 Canada; 3 Department of Biology, McMaster University, 1280 Main St. West, Hamilton, ON L8S 4K1 Canada; 4 Department of Molecular, Cell and Systems Biology, University of California, Riverside, 900 University Ave., Riverside, CA 92521 USA

The original article can be found online at 10.1038/s41598-024-62974-4.

Correction to: *Scientific Reports*10.1038/s41598-024-62974-4, published online 03 June 2024

The original version of this Article contained an error in the spelling of the author Andrew Zogby which was incorrectly given as Andrew Zobgy.

The original Article has been corrected.

